# TWEAK enhances TGF-β-induced epithelial-mesenchymal transition in human bronchial epithelial cells

**DOI:** 10.1186/s12931-015-0207-5

**Published:** 2015-04-08

**Authors:** Yukinari Itoigawa, Norihiro Harada, Sonoko Harada, Yoko Katsura, Fumihiko Makino, Jun Ito, Fariz Nurwidya, Motoyasu Kato, Fumiyuki Takahashi, Ryo Atsuta, Kazuhisa Takahashi

**Affiliations:** Department of Respiratory Medicine, Juntendo University Faculty of Medicine and Graduate School of Medicine, 3-1-3 Hongo, Bunkyo-ku, Tokyo, 113-8431 Japan; Research Institute for Diseases of Old Ages, Juntendo University Faculty of Medicine and Graduate School of Medicine, Tokyo, Japan; Atopy (Allergy) Research Center, Juntendo University Faculty of Medicine and Graduate School of Medicine, Tokyo, Japan

**Keywords:** Bronchial epithelial cells, TGF-β, TWEAK, Epithelial-mesenchymal transition (EMT), Smad2, p38 MAPK, NF-κB, ZEB2

## Abstract

**Background:**

Chronic airway inflammatory disorders, such as asthma, are characterized by airway inflammation and remodeling. Chronic inflammation and damage to the airway epithelium cause airway remodeling, which is associated with improper epithelial repair, and is characterized by elevated expression of transforming growth factor-β (TGF-β). Epithelial-mesenchymal transition (EMT) is an important mechanism during embryonic development and tissue remodeling whereby epithelial cells gain the capacity to increase motility by down-regulation of epithelial markers and up-regulation of mesenchymal markers. TGF-β is a central inducer of EMT, and TGF-β-induced EMT is enhanced by pro-inflammatory cytokines, including tumor necrosis factor-α (TNF-α) and interleukin-1β. We investigated whether the pro-inflammatory cytokine TWEAK (TNF-like weak inducer of apoptosis) enhanced TGF-β1-induced EMT in the human bronchial epithelial cell line BEAS-2B.

**Methods:**

Quantitative RT-PCR and western blotting were used to define alterations in epithelial and mesenchymal marker expression in BEAS-2B cells. The cells were assessed for 48 h after stimulation with TGF-β1 alone or in combination with TWEAK.

**Results:**

TGF-β1 induced spindle-like morphology and loss of cell contact, and reduced the expression of epithelial marker E-cadherin and increased the expression of mesenchymal markers N-cadherin and vimentin. Our data, for the first time, show that TWEAK reduced the expression of E-cadherin, and that co-treatment with TGF-β1 and TWEAK enhanced the TGF-β1-induced features of EMT. Moreover, hyaluronan synthase 2 expression was up-regulated by a combination with TGF-β1 and TWEAK, but not TNF-α. We also demonstrated that the Smad, p38 MAPK, and NF-κB signaling pathways, and the transcriptional repressor ZEB2 might mediate N-cadherin up-regulation by TGF-β1 in combination with TWEAK.

**Conclusions:**

These findings suggest that the pro-inflammatory cytokine TWEAK and TGF-β1 have synergistic effects in EMT and may contribute to chronic airway changes and remodeling.

**Electronic supplementary material:**

The online version of this article (doi:10.1186/s12931-015-0207-5) contains supplementary material, which is available to authorized users.

## Background

Although mechanisms of chronic airway inflammatory disorders, including asthma, are not fully clarified, they are all characterized by airway inflammation and remodeling [1,2]. Airway remodeling features basement membrane thickening, excessive deposition of extracellular matrix (ECM), mucus cell metaplasia, epithelial shedding, angiogenesis, inflammatory cell infiltration, and smooth-muscle cell and lung fibroblast proliferation [3]. Epithelial repair consists of a complex cascade of events that starts immediately after injury and leads to the organized repopulation of epithelial cells. Persistent epithelial damage might be the result of incomplete repair where any component of this cascade does not work properly. Therefore, the combination of chronic epithelial injury and prolonged epithelial repair leads to overproduction of pro-fibrotic growth factors and the proliferation and differentiation of fibroblasts into myofibroblasts. The exact mechanisms of the origin of mesenchymal cell expansion are unknown, although the expansion of the resident fibroblast population and recruitment of fibrocytes from circulation have both been implicated [4]. A recent report has suggested that epithelial cells can contribute to the local fibroblast pool during a process of molecular reprogramming called epithelial-mesenchymal transition (EMT) [5]. EMT is also an essential component of embryonic development, tissue remodeling, and wound repair [6]. During this transition, epithelial cells acquire the capacity to increase motility through down-regulating epithelial markers, such as E-cadherin, and increasing expression of mesenchymal proteins, such as N-cadherin and vimentin [7,8]. EMT can be induced by such growth factors as transforming growth factor-β (TGF-β), which is secreted by various cells including airway epithelial cells and infiltrating immune cells [9-11]. It has been reported that house dust mite and some pro-inflammatory cytokines, including tumor necrosis factor-α (TNF-α) and interleukin-1β (IL-1β), can enhance TGF-β-induced EMT in bronchial epithelial cells [12-14]. In this regard, pro-inflammatory cytokines can contribute to airway remodeling in chronic inflammatory disorders.

TNF-like weak inducer of apoptosis (TWEAK) and its receptor fibroblast growth factor-inducible 14 (Fn14) have emerged as a ligand/receptor pair in the TNF superfamily. Although the TWEAK/Fn14 interaction contributes to pro-inflammatory effects and tissue remodeling [15,16] and high TWEAK expression was observed in the skin of patients with atopic dermatitis as compared with healthy subjects [17], the exact mechanisms that underlie TWEAK-mediated airway remodeling have not been elucidated. In the present study, we investigated whether TWEAK induced EMT and enhanced TGF-β-induced EMT. Our data, for the first time, show that TWEAK down-regulates E-cadherin expression and accelerates TGF-β1-induced EMT in bronchial epithelial cells in an *in vitro* culture model. Moreover, hyaluronan synthase 2 expression was up-regulated by a combination with TGF-β1 and TWEAK, but not TNF-α. We also demonstrated that Smad-dependent and Smad-independent signaling pathways, including p38 mitogen-activated protein kinase (MAPK) and nuclear factor κB (NF-κB), and the transcriptional repressor ZEB2 might mediate N-cadherin up-regulation by TGF-β1 in combination with TWEAK. These findings suggest that TWEAK has synergistic effects with TGF-β1-induced features of EMT and may contribute to chronic airway changes and remodeling.

## Materials and methods

### Reagents

Recombinant soluble human TGF-β1 and TWEAK were from Peprotech (Rocky Hill, NJ, USA). Recombinant soluble human TNF-α was obtained from eBioscience (San Diego, CA, USA). Purified anti-α-tublin and anti-human Vimentin (V9) monoclonal antibodies (mAbs) SB431542 and AG1478 were from Sigma Chemicals (St. Louis, MO, USA). Anti-human E-cadherin (HECD-1) was from Takara (Tokyo, Japan). N-cadherin and anti-EGFR mAbs were from BD Biosciences (San Jose, CA, USA). Anti-phospho-EGFR (pY845) mAbs was from abcam (Cambridge, UK). Anti-Smad2/3, anti-phospho-Smad2 (Ser465/467), anti-extracellular signal-regulated kinase (ERK), anti-phospho-ERK (Thr202/Tyr204), anti-p38 MAPK, anti-phospho-p38 MAPK (Thr180/Tyr182), anti-Akt, anti-phospho-NF-κB p65 (Ser536) polyclonal antibodies, and anti-ZO-1, anti- Jun N-terminal kinase (JNK), anti-phospho-JNK (Thr183/Tyr185), anti-phospho-Akt (Ser473), and anti-NF-κB mAbs were obtained from Cell Signaling Technology (Beverly, MA, USA). SB202190, SP600125, LY294002, and BAY11-7082 were from Wako Chemicals (Osaka, Japan). AZD6244 was from Selleckchem (Houston, TX, USA). Bronchial epithelial growth medium (BEGM) was purchased from Cambrex (East Rutherford, NJ, USA).

### Cell culture

The SV40-transformed normal human bronchial epithelial cell line BEAS-2B was purchased from ATCC (Rockville, MD, USA). Primary normal human bronchial epithelial (NHBE) cells were purchased from Cambrex. Cells were grown on collagen I-coated flasks or plates (Asahi Techno Glass, Chiba, Japan). BEAS-2B cells and NHBE cells were cultured in complete BEGM, which consists of bronchial epithelial basal medium (BEBM) supplemented with insulin (5 μg/ml), hydrocortisone (0.5 μg/ml), transferrin (10 μg/ml), triiodothyronine (6.5 ng/ml), epinephrine (0.5 μg/ml), human EGF (0.5 ng/ml), retinoic acid (0.1 ng/ml), gentamycin (50 μg/ml), and bovine pituitary extract (52 μg/ml). The cultured media were changed to fresh BEBM without growth factor and serum with or without recombinant soluble human TGF-β1 (10 ng/ml), TNF-α (10 ng/ml), or different concentrations of TWEAK (1-100 ng/ml), which was as described in the Results.

### RNA Isolation and quantitative RT-PCR

Total cell RNA was isolated from bronchial epithelial cells using the RNeasy plus mini kit (Qiagen, Valencia, CA, USA) with DNase treatment, followed by cDNA synthesis using the First-Strand cDNA Synthesis kit (GE Healthcare, Little Chalfont, Buckinghamshire, UK) according to the manufacturer’s instructions. Fast SYBR Green Master Mix (Applied Biosystems, Foster City, CA, USA) and an ABI 7500 Fast real-time PCR instrument (Applied Biosystems, Warrington, UK) were used for quantitative real-time reverse transcription-PCR (qRT-PCR) with the gene specific primer pairs listed in Table 1. For data analysis, the comparative threshold cycle (CT) value for GAPDH was used to normalize loading variations in the real-time PCRs. A ΔΔC_T_ value then was obtained by subtracting control ΔC_T_ values from the corresponding experimental ΔC_T_ values. The ΔΔC_T_ values were converted to fold differences as compared to the control by raising two to the ΔΔCT power.

**Table 1:** **Primer sequences for real-time PCR**

**Target**	**Sense primer(5′→3′)**	**Antisense primer(5′→3′)**
E-cadherin	ACCTCCATCACAGAGGTTCC	GAAGCCGAGGTTTTAACTGC
Zo-1	GAGGACCAGCTGAAGGACAG	TGCATTTTTCCCACTTTTCC
N-cadherin	AGCCAACCTTAACTGAGGAGT	GGCAAGTTGATTGGAGGGATG
Vimentin	AATTGCAGGAGGAGATGCTT	GAGACGCATTGTCAACATCC
Tenascin-C	CTGGACTTGCTCCCAGCATC	CCAGGAAACTGTGAACCCGTA
HAS-2	GTGATGACAGGCATCTCA	GCGGGAAGTAAACTCGA
Fibronectin	GAAGCCGAGGTTTTAACTGC	ACCCACTCGGTAAGTGTTCC
Snail	CTCTTTCCTCGTCAGGAAGC	GGCTGCTGGAAGGTAAACTC
Slug	CCAAACTACAGCGAACTGGA	GTGGTATGACAGGCATGGAG
Twist	CCGGAGACCTAGATGTCATTG	CCACGCCCTGTTTCTTTG
ZEB1	AACCCAACTTGAACGTCACA	ATTACACCCAGACTGCGTCA
ZEB2	CAGACCGCAATTAACAATGG	TACTCCTCGATGCTGACTGC
GAPDH	GGTCTCCTCTGACTTCAACA	GTGAGGGTCTCTCTCTTCCT

### Preparation of cell lysates and western blotting

Whole cell lysates were prepared at the indicated time points using RIPA buffer (50 mM Tris-HCl, pH 8.0, 150 mM NaCl, 1% Nonidet P-40, 0.5% deoxycholate, 0.1% sodium dodecyl sulfate, 25 mM β-glycerophosphate, 1 mM sodium orthovanadate, 1 mM sodium fluoride, 1 mM phenylmethylsulfonyl fluoride, 1 μg/ml aprotinin, and 1 μg/ml leupeptin). Protein concentration was determined by the Bradford assay. Equal amounts of whole cell lysates (10-20 μg) were separated by 8% or 10% SDS-PAGE and blotted onto polyvinylidene difluoride membranes. After blocking with 5% skim milk, blots were incubated overnight with the indicated primary antibodies. Anti-E-cadherin, anti-ZO-1, anti-EGFR, anti-phospho-EGFR, anti-JNK, anti-phospho-JNK, anti-phospho-Akt, anti-NF-κB mAbs, and anti-α-tublin mAbs, and anti-Smad2/3, anti-phospho-Smad2, anti-ERK, anti-phospho-ERK, anti-p38 MAPK, anti-phospho-p38 MAPK, anti-Akt, and anti-phospho-NF-κB p65 polyclonal antibodies were used at 1/1000 dilution. Anti-N-cadherin and Vimentin mAbs were used at 1/500 and 1/4000 dilution, respectively. Membranes were then incubated with appropriate horseradish peroxidase-conjugated secondary antibodies (used at 1/4000 dilution, GE Healthcare), followed by detection with ECL Plus (GE Healthcare). All dilutions were in TBST (Tris-buffered saline containing 0.1% Tween-20) supplemented with 5% bovine serum albumin. Densitometry of western blot signals acquired with a Fuji LAS-4000 fluorescence imager (Fujifilm Corporation, Tokyo, Japan) with a linearity of 4 orders of magnitude was performed using NIH ImageJ image analysis software.

### RNA interference assay

Lipofectamine RNAiMAX transfection agent and oligonucleotides used in this study were purchased or custom-synthesized by Life Technologies, Inc. (Grand Island, NY, USA) and the sense and antisense sequences are listed in Table 2. BEAS-2B cells were transfected with 20 nM small interfering RNA (siRNA) duplexes using Lipofectamine RNAiMAX according to the manufacturer’s instructions. Briefly, siRNA duplexes and Lipofectamine RNAiMAX were incubated in Opti-MEM I reduced serum medium (Opti-MEM) (Life Technologies, Inc.) at room temperature for 15 min. The cells were transfected with the siRNA/Lipofectamine RNAiMAX complexes and seeded at a density of 2.5 × 10^5^ cells on 6-well plates. After incubation for 24 h at 37°C, the transfected cells were treated with the absence (control) or presence of TGF-β1 (10 ng/ml), TNF-α (10 ng/ml), or TWEAK (100 ng/ml) for 48 h. Knockdown efficacy was evaluated with quantitative real-time RT-PCR at the designated time points.

**Table 2:** **Oligonucleotide design**

**Target**	**Sense primer(5′ → 3′)**	**Antisense primer(5′ → 3′)**
ZEB1 #1	AAACAGAGGACUCAGGCUUCUCAGC	GCUGAGAAGCCUGAGUCCUCUGUUU
ZEB1 #2	UUAAGCAUGGAACACUGUUCUGGUC	GACCAGAACAGUGUUCCAUGCUUAA
ZEB2 #1	UCAUACACUCCAAACAGCUUCUCUU	AAGAGAAGCUGUUUGGAGUGUAUGA
ZEB2 #2	GAAAGAGAAGCUACGUACUUUAAUA	UAUUAAAGUACGUAGCUUCUCUUUC

### Immunofluorescence staining for E-cadherin and N-cadherin

Confluent monolayers grown on Nunc Lab-Tek chamber slides (Thermo Scientific, Rockford, IL, USA) were fixed with 4% paraformaldehyde for 10 min at room temperature and stained with anti-E cadherin mAbs (used at 1/1000 dilution) or anti-N cadherin mAbs (used at 1/500 dilution), followed by the secondary antibody (goat anti-mouse IgG conjugated with Alexa594, used at 1/4000 dilution, Life Technologies). Nuclei were stained with 4′, 6-diamidino-2-phenylindole (DAPI) (Sigma). Fluorescence images were captured using an epifluorescence microscope (Axioplan 2 imaging; Zeiss, Jena, Germany) and image acquisition was performed with Axiovision 4.0 software (Zeiss).

### Monolayer wound-healing assay

We have established this method previously [18]. Briefly, BEAS-2B and NHBE cells were grown in 6-well plate and then placed upon confluence in the growth factor–free BEBM. The circular wounds (~2.0 mm2) or linear wounds were made in the confluent monolayer using a 20-μl pipette tip (4 circular wounds or 1 × 1 linear wounds in each well). In each experiment, one well was used as a negative control with no treatment. The wounds were imaged 0 and 48 hours after wound creation using a Nikon Eclipse TE200 inverted microscope equipped with a Nikon Coolpix E995. Corresponding wound areas were determined using ImagePro Plus and the remaining wound areas were calculated as a percentage of area at time 0.

### Flow cytometric analysis

BEAS-2B cells (0.5–1.0 × 10^6^) were incubated with Phycoerythrin-conjugated anti-Fn14 mAbs (eBioscience, San Diego, CA, USA). After washing with PBS twice, the stained cells (live gated on the basis of forward and side scatter profiles and propidium iodide exclusion) were analyzed with a FACSCalibur system (BD Biosciences, Mountain View, CA, USA), and data were processed by using the CellQuest program (BD Biosciences).

### Proliferation assay

An equivalent number of cells (5.0 × 10^3^/well) was flat-bottomed 96-well microtiter plate and allowed to adhere for 24 h at 37°C. After washing with PBS twice, cells were incubated with fresh BEBM without growth factor and serum with or without recombinant soluble human TGF-β1 (10 ng/ml), TNF-α (10 ng/ml), or different concentrations of TWEAK (1-100 ng/ml) for 48 h at 37°C. After treatment, 10 μl Cell counting Kit 8 solution (Dojindo Laboratories, Kumamoto, Japan) was added to each well, and the 96-well plates were continuously incubate at 37°C for 2 h. Absorbance was read at 450 nm on a Microplate reader with Microplate manager (Bio-Rad Laboratories, Hercules, CA, USA).

### Statistical analysis

Comparisons between multiple groups were made by one-way ANOVA with Tukey’s multiple comparisons test using the program GraphPad Prism version 6 (GraphPad Software, San Diego, CA, USA). Differences were considered to be statistically significant when *p* values were 0.05 or less.

## Results

### TWEAK regulates E-cadherin expression and enhances TGF-β1-induced EMT in bronchial epithelial cells

**Figure 1:**
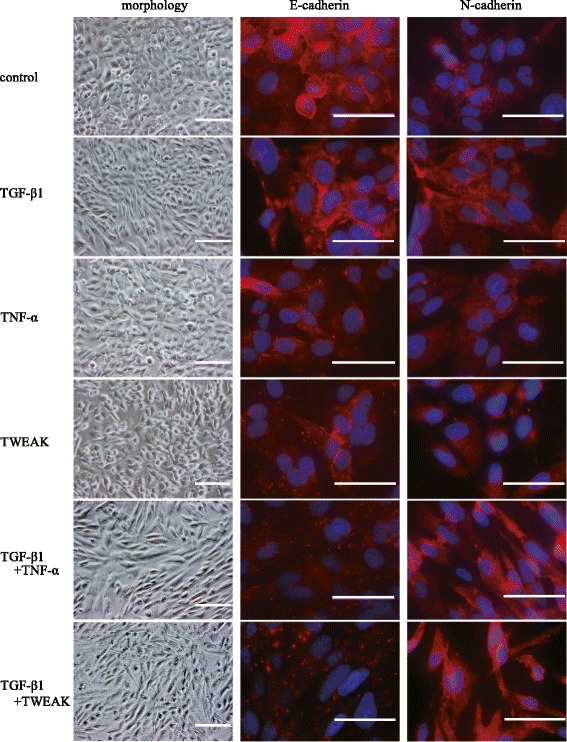
**Changes of morphology and E-cadherin and N-cadherin protein levels in BEAS-2B cells upon TGF-β1 and TWEAK treatment.** Confluent monolayers of BEAS-2B cells were cultured for 48 h in the absence (control) or presence of TGF-β1 (10 ng/ml), TNF-α (10 ng/ml), TWEAK (100 ng/ml), or TGF-β1 in combination with TNF-α or TWEAK. E-cadherin and N-cadherin (red) was assessed by immunofluorescent staining. The nuclei were stained with DAPI for DNA (blue). Scale bar 40 μm.

**Figure 2:**
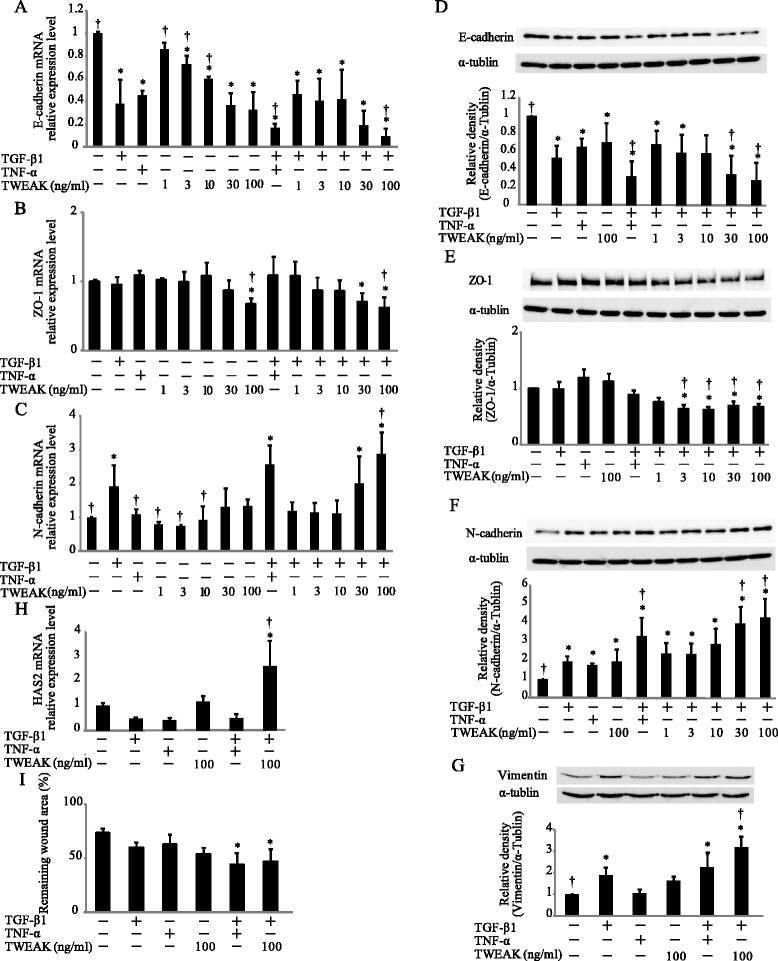
**Cooperative induction of EMT in BEAS-2B cells by TGF-β1 and TWEAK.** Confluent monolayers of BEAS-2B cells were cultured for 48 h in the absence (control) or presence of TGF-β1 (10 ng/ml), TNF-α (10 ng/ml), different concentrations of TWEAK (1-100 ng/ml), or TGF-β1 in combination with TNF-α or TWEAK (1-100 ng/ml) as indicated. The levels of E-cadherin **(A)**, ZO-1 **(B)**, N-cadherin **(C)**, and HAS2 **(H)** mRNA were analyzed by qRT-PCR. Expression levels were normalized to the housekeeping gene GAPDH and calculated as fold induction in comparison to the control. Whole cell lysates were immunoblotted for E-cadherin (**D**, upper), ZO-1 (**E**, upper), N-cadherin (**F**, upper), or vimentin (**G**, upper). All membranes were re-probed with anti-α-tublin antibody to confirm equal loading. The density of each band was normalized with α-tubulin and quantified by densitometry using NIH ImageJ software (**D-G**, lower). The capability of cell migration was assessed using a wound-healing assay **(I)**. Data represent the means ± SD of three independent experiments. ^*^ *p* < 0.05 compared with the untreated culture. ^†^ *p* < 0.05 compared with TGF-β1 alone.

A previous report demonstrated that BEAS-2B cells express TWEAK receptor Fn14 [19]. We also confirmed by flow cytometric analysis that this receptor existed on BEAS-2B cells (Additional file 1: Figure S1). We first investigated whether exogenous TWEAK could induce EMT and enhance TGF-β1-induced EMT similarly to TNF-α in BEAS-2B cells. Confluent monolayers of BEAS-2B cells were treated with or without TGF-β1 (10 ng/ml), TNF-α (10 ng/ml), TWEAK (100 ng/ml), or TGF-β1 in combination with TNF-α or TWEAK. We decided to use a dose of 100 ng/ml TWEAK as a stimulus because TWEAK in a similar concentration was used in most previous reports [16,19-22]. Phase contrast microscopy showed that cells treated with TNF-α or TWEAK in combination with TGF-β1, compared with control and TGF-β1 alone, exhibited spindle-like morphology and loss of cell contact (Figure 1). Immunofluorescent staining of BEAS-2B cells, which usually express high levels of E-cadherin, revealed dramatically reduced expression of this adherens junction protein after 48 h of treatment with TGF-β1 alone, TNF-α, or TWEAK (Figure 1). Moreover, TGF-β1 in combination with TNF-α or TWEAK more strongly down-regulated the expression of E-cadherin by immunohistochemistry as compared with treatment with TGF-β1 alone (Figure 1). Furthermore, TGF-β1 induced N-cadherin expression and TGF-β1 in combination with TNF-α or TWEAK more strongly induced N-cadherin expression in immunofluorescent staining of BEAS-2B cells (Figure 1). Additionally, total RNAs and whole cell lysates were isolated from the cells at 48 h after treatment with or without TGF-β1 (2 or 10 ng/ml), TNF-α (10 ng/ml), different concentrations of TWEAK (1-100 ng/ml), or TGF-β1 in combination with TNF-α or TWEAK (1-100 ng/ml). The levels of E-cadherin, ZO-1 and N-cadherin mRNA and protein were analyzed by quantitative real-time PCR and western blotting, respectively. Compared with controls, BEAS-2B cells treated with TGF-β1, TNF-α, or a combination of TGF-β1 and TNF-α exhibited down-regulation of mRNA and protein expression of E-cadherin as an epithelial marker (Figures 1, 2A and 2D), and up-regulation of N-cadherin expression as a mesenchymal marker (Figures 1, 2C and 2F). TWEAK alone and in combination with TGF-β1 also down-regulated the expression of E-cadherin (Figures 1, 2A and 2D, and Additional file 2: Figure S2A). Moreover, TWEAK in combination with TGF-β1 also down-regulated the expression of ZO-1 as an epithelial marker (Figure 2B and 2E) and up-regulated the expression of N-cadherin (Figure 2C and 2F and Additional file 2: Figure S2B) in a dose-dependent manner. Furthermore, the protein levels of another mesenchymal marker, vimentin, were enhanced by TGF-β1 alone, TWEAK alone, and by combinations of TGF-β1 and TNF-α or TWEAK (Figure 2G). Additionally, expression of the major ECM-related genes was examined. Additional file 3: Figure S3 showed that a combination of TGF-β1 and TNF-α, but not TWEAK, enhanced TGF-β1-induced mRNA expression of Tenascin-C and fibronectin. Expression of another ECM constituent, hyaluronan synthase 2 (HAS2), which is one of the hyaluronan synthase enzyme, increased by TWEAK and the combinations of TGF-β1 and TWEAK, but not TNF-α (Figure 2H).

We next examined cell migration, which was assessed using a wound-healing assay, as important hallmarks of EMT. Compared with controls, BEAS-2B cells treated with a combination of TGF-β1 and TNF-α or TWEAK exhibited significant migration in wound-healing assay (Figure 2I and Additional file 4: Figure S4). However, the treatment with TGF-β1, TNF-α, TWEAK, or TGF-β1 in combination with TNF-α or TWEAK had no effect on proliferation of BEAS-2B cells (Additional file 5: Figure S5).

**Figure 3:**
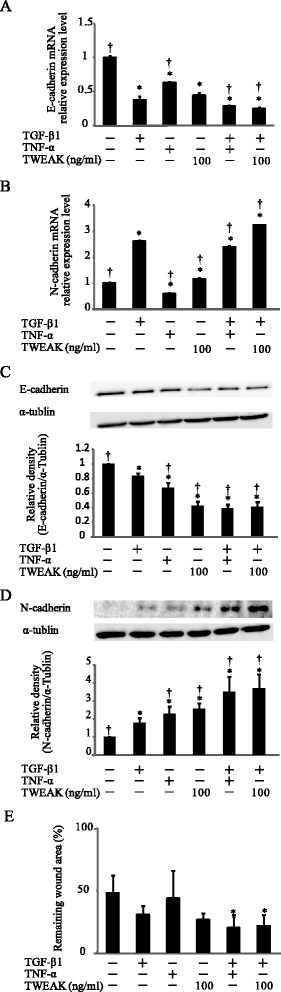
**Cooperative induction of EMT in NHBE cells by TGF-β1 and TWEAK.** Confluent monolayers of NHBE cells were cultured for 48 h in the absence (control) or presence of TGF-β1, TNF-α, TWEAK, or TGF-β1 in combination with TNF-α or TWEAK, as indicated. The levels of E-cadherin **(A)** and N-cadherin **(B)** mRNA were analyzed by qRT-PCR. Expression levels were normalized to the housekeeping gene GAPDH and calculated as fold induction in comparison to the control. Whole cell lysates were immunoblotted for E-cadherin (**C**, upper) and N-cadherin (**D**, upper). All membranes were re-probed with anti-α-tublin antibody to confirm equal loading. The density of each band was normalized with α-tubulin and quantified by densitometry using NIH ImageJ software (**C** and **D**, lower). The capability of cell migration was assessed using a wound-healing assay **(E)**. Data represent the means ± SD of three independent experiments. ^*^ *p* < 0.05 compared with the untreated culture. ^†^ *p* < 0.05 compared with TGF-β1 alone.

Further we investigated whether TWEAK could down-regulate E-cadherin expression and enhance TGF-β1-induced EMT in primary cultures of NHBE cells. In NHBE cells, TWEAK down-regulated E-cadherin expression and enhanced TGF-β1-induced E-cadherin down-regulation and N-cadherin up-regulation and exhibited significant migration in wound-healing assay (Figure 3). These results indicate that not only TNF-α, but also TWEAK can down-regulate E-cadherin expression and enhance TGF-β1-induced EMT in bronchial epithelial cells.

### Smad-dependent and Smad-independent signaling pathways are required for N-cadherin up-regulation, but not E-cadherin down-regulation, caused by a combination of TGF-β1 and TWEAK

**Figure 4:**
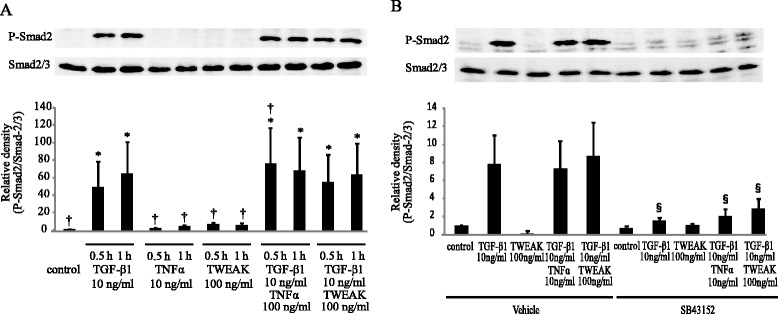
**TWEAK had no additional effect on TGF-β1-induced Smad2 phosphorylation.** Confluent monolayers of BEAS-2B cells were cultured for the indicated time points in the absence (conrol or DMSO as vehicle) or presence of SB43125 (10 μM) and treated with TGF-β1 (10 ng/ml), TNF-α (10 ng/ml), TWEAK (100 ng/ml), or TGF-β1 in combination with TNF-α or TWEAK, as indicated. Whole cell lysates were prepared at the indicated time points **(A)** and 1 h **(B)** after treatment. Phosphorylation of Smad2 was examined by western blotting. Densitometry of pSmad2 signals was normalized against total Smad2 signals. Data represent the means ± SD of three independent experiments. ^*^ *p* < 0.05 compared with the untreated culture. ^†^ *p* < 0.05 compared with TGF-β1 alone. ^§^ *p* < 0.05 compared with vehicle.

**Figure 5:**
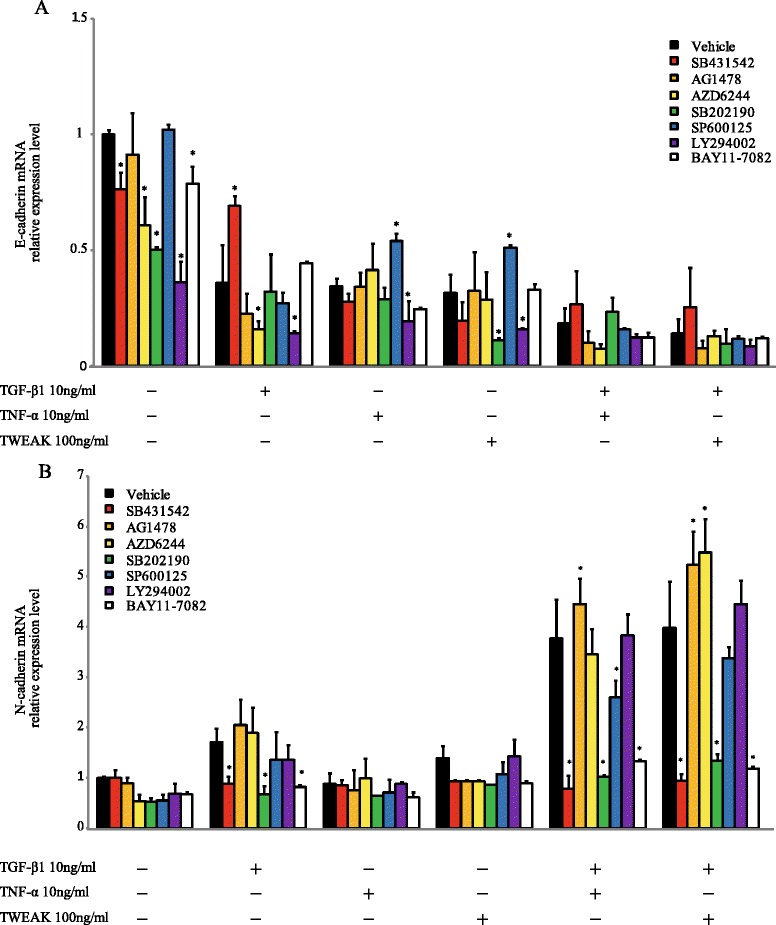
**The combination of TGF-β1 and TWEAK-induced N-cadherin up-regulation required Smad, p38 MAPK, and NF-κB signaling pathways.** Confluent monolayers of BEAS-2B cells were cultured for 48 h in the absence (DMSO as vehicle) or presence of SB43125 (10 μM), AG1478 (1 μM), AZD6244 (5 μM), SB202190 (5 μM), SP600125 (5 μM), LY294002 (5 μM), or BAY11-7082 (2.5 μM) and treated with TGF-β1 (10 ng/ml), TNF-α (10 ng/ml), TWEAK (100 ng/ml), or TGF-β1 in combination with TNF-α or TWEAK, as indicated. The levels of E-cadherin **(A)** and N-cadherin **(B)** mRNAs were analyzed by qRT-PCR. Expression levels were normalized to the housekeeping gene GAPDH and calculated as fold induction in comparison to vehicle. Data represent the means ± SD of three independent experiments. ^*^ *p* < 0.05 compared with vehicle.

Next, we investigated whether Smad-dependent and Smad-independent signaling pathways are required for the TWEAK-enhanced EMT that was induced by TGF-β1. Confluent monolayers of BEAS-2B cells were cultured for 0.5 h or 1 h with or without TGF-β1 (10 ng/ml), TWEAK (100 ng/ml), or TGF-β1 in combination with TWEAK. Although TGF-β1 increased Smad2 phosphorylation, TWEAK had no additional effect on it (Figure 4). Additionally, Smad2 phosphorylation and N-cadherin up-regulation induced by TGF-β1 or a combination of TGF-β1 and TNF-α or TWEAK was completely inhibited by SB431542 (10 μM), an inhibitor of the TGF-β receptor type 1 (TβRI) kinase, which is known to be an activator of the Smad pathway (Figures 4B and 5B). In contrast, SB431542 was not found to attenuate TNF-α or TWEAK-mediated E-cadherin down-regulation, although down-regulation of E-cadherin induced by TGF-β1 was attenuated by SB431542 (Figure 5A). These results suggest that Smad-dependent pathways are required for N-cadherin up-regulation by TGF-β1 or a combination of TGF-β1 and TWEAK, but are not required for TWEAK-mediated E-cadherin down-regulation. Conversely, additional Smad-independent pathways may be required for TWEAK-enhanced TGF-β1-induced EMT.

TGF-β1 also activates Smad-independent pathways such as ERK, p38 MAPK, JNK, phosphatidylinositol 3-kinase (PI3K)/Akt, and NF-κB [23]. To explore the involvement of Smad-independent pathways in TWEAK-induced E-cadherin down-regulation and TWEAK-enhanced TGF-β1-induced EMT, we examined whether a combination with TGF-β1 and TWEAK could activate Smad-independent pathways. TGF-β1 in combination with TNF-α or TWEAK led to activation of the Smad-independent pathways via phosphorylation of ERK, p38 MAPK, JNK, PI3K/Akt, NF-κB, and epidermal growth factor receptor (EGFR) (Additional file 6: Figure S6). TWEAK alone also induced the phosphorylation of ERK, JNK, PI3K/Akt, NF-κB, and EGFR. Especially, TWEAK induced the phosphorylation of JNK to higher levels than those induced by a combination with TGF-β1 and TNF-α. Furthermore, these phosphorylations were inhibited by each inhibitor, including AZD6244 (RAF/MEK/ERK pathway inhibitor), SB202190 (p38 MAPK inhibitor), SP600125 (JNK inhibitor), LY294002 (PI3K inhibitor), BAY11-7082 (NF-κB inhibitor), or AG1478 (EGFR inhibitor).

**Figure 6:**
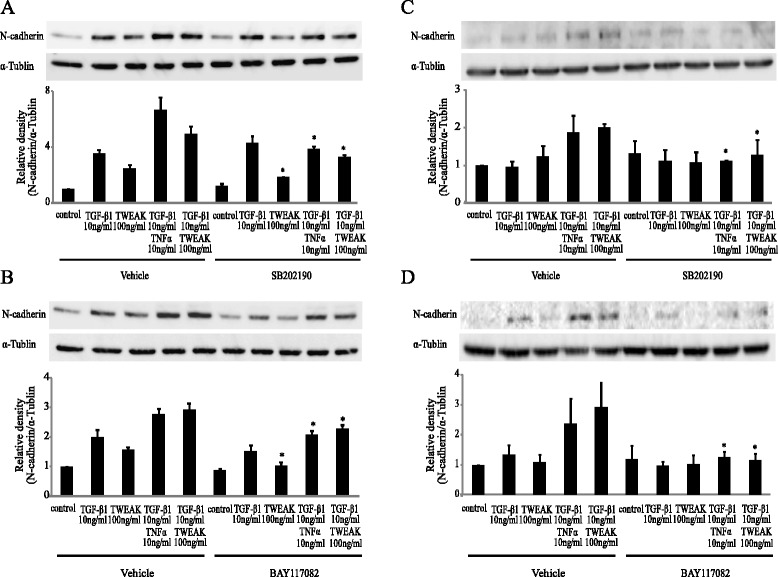
**The combination of TGF-β1 and TWEAK-induced N-cadherin up-regulation required p38 MAPK and NF-κB signaling pathways.** Confluent monolayers of BEAS-2B cells (**A** and **B**) and NHBE cells (**C** and **D**) were cultured for 48 h in the absence (DMSO as vehicle) or presence of SB202190 (5 μM) (**A** and **C**) and BAY11-7082 (2.5 μM) (**B** and **D**) and treated with TGF-β1, TWEAK, or TGF-β1 in combination with TNF-α or TWEAK, as indicated. Whole cell lysates were immunoblotted for N-cadherin (upper). The membranes for N-cadherin were re-probed with anti-α-tublin antibody to confirm equal loading. The density of N-cadherin was normalized with α-tubulin and quantified by densitometry (lower). Data represent the means ± SD of three independent experiments. ^*^ *p* < 0.05 compared with vehicle.

We next investigated whether inhibitors for Smad-independent pathways had any effect on TWEAK-mediated EMT. Confluent monolayers of BEAS-2B cells were cultured for 48 h with or without TGF-β1, TNF-α, TWEAK, or TGF-β1 in combination with TNF-α or TWEAK, and were treated with or without SB431542, AG1478, AZD6244, SB202190, SP600125, LY294002, or BAY11-7082. As shown in Figure 5A, these inhibitors without SB431542 had no apparent effect on the E-cadherin down-regulation that was induced by TGF-β1, TNF-α, TWEAK, and the combinations with TGF-β1. In contrast, N-cadherin up-regulation induced by TGF-β1 or a combination of TGF-β1 and TNF-α or TWEAK was completely inhibited by SB431542, SB202190 and BAY11-7082, although AG1478, AZD6244, SP600125, and LY294002 had no effect on N-cadherin up-regulation (Figure 5B). Moreover, we examined whether SB202190 and BAY11-7082 could inhibit the protein levels of N-cadherin which were induced by the combination of TGF-β1 and TWEAK. Figure 6 and Additional file 7: Figure S7 showed that TGF-β1 in combination with TNF-α- or TWEAK-induced N-cadherin expressions was partially inhibited by SB202190 and BAY11-7082 in BEAS-2B and NHBE cells, but not SP600125 in BEAS-2B cells. These results suggest that Smad-dependent pathways and p38 MAPK and NF-κB, which are Smad-independent pathways, play an important role in N-cadherin up-regulation by a combination of TGF-β1 and TWEAK in bronchial epithelial cells. However, p38 MAPK and NF-κB were not required for TWEAK-induced and TWEAK-enhanced E-cadherin down-regulation.

### TWEAK induces mRNA expression of transcriptional repressors ZEB1 and ZEB2 in BEAS-2B cells

**Figure 7:**
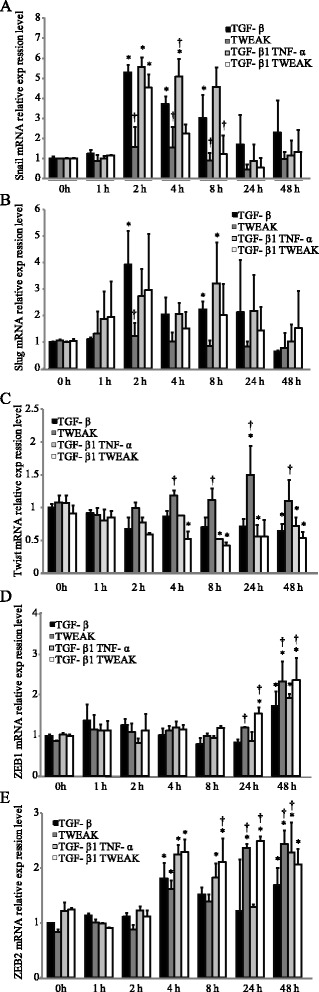
**TWEAK induces mRNA expression of transcriptional repressors ZEB1 and ZEB2.** Confluent monolayers of BEAS-2B cells were cultured for the indicated time points in the absence (control) or presence of TGF-β1 (10 ng/ml), TNF-α (10 ng/ml), TWEAK (100 ng/ml), or TGF-β1 in combination with TNF-α or TWEAK. The levels of Snail **(A)**, Slug **(B)**, TWIST **(C)**, ZEB1 **(D)**, and ZEB2 **(E)** mRNAs were analyzed by qRT-PCR. Expression levels were normalized to the housekeeping gene GAPDH and calculated as fold induction in comparison to 0 h. Data represent the means ± SD of three independent experiments. ^*^ *p* < 0.05 compared with 0 h. ^†^ *p* < 0.05 compared with TGF-β1 alone.

Recently, it was reported that EMT is controlled by a group of transcriptional repressors [24,25]. The zinc-finger-containing proteins Snail, Slug, ZEB1, ZEB2 (also denoted SIP1), and the helix-loop-helix transcription factors Twist are important transcriptional repressors that bind to the E-boxes in the promoter of the E-cadherin gene and actively repress its expression [24,25]. To examine whether transcriptional repressors mediated the TWEAK-enhanced EMT that was induced by TGF-β1 in BEAS-2B cells, total RNA was extracted from stimulated monolayers of BEAS-2B cells with or without TGF-β1, TWEAK, or TGF-β1 in combination with TNF-α or TWEAK. TGF-β1 significantly increased the expression of Snail and Slug mRNA (Figure 7A and B). TWEAK treatment with TGF-β1 at 8 h significantly repressed the TGF-β1-induced Snail mRNA expressions (Figure 7A). Although TWEAK significantly increased the expression of Twist mRNA, TWEAK in combination with TGF-β1 repressed the expression of Twist mRNA (Figure 7C). TGF-β1, TWEAK, and a combination of TGF-β1 and TNF-α or TWEAK significantly increased mRNA expression of ZEB1 and ZEB2 (Figure 7D and E), suggesting that ZEB1/2 may be required for TWEAK stimulation in bronchial epithelial cells.

### Knockdown of ZEB2, but not of ZEB1, reduces TWEAK-enhanced N-cadherin mRNA expression in BEAS-2B cells

**Figure 8:**
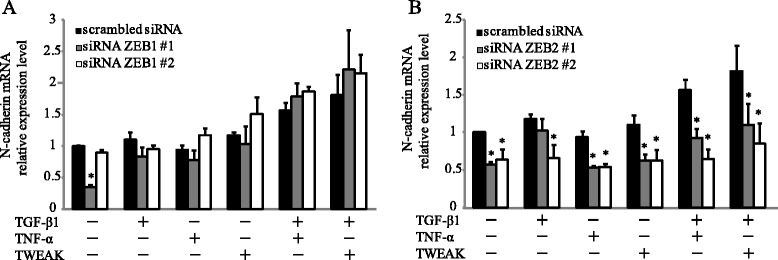
**ZEB2 knockdown reduces TWEAK-enhanced N-cadherin mRNA expression.** Total RNA was extracted from BEAS-2B cells treated with or without TGF-β1 (10 ng/ml), TNF-α (10 ng/ml), TWEAK (100 ng/ml), or TGF-β1 in combination with TNF-α or TWEAK for 48 h at 72 h after transfection with control siRNA, ZEB1 siRNA **(A)**, or ZEB2 siRNA **(B)**. The levels of N-cadherin mRNA were analyzed by qRT-PCR. Expression levels were normalized to the housekeeping gene GAPDH and calculated as fold induction in comparison to control. Data represent the means ± SD of three independent experiments. ^*^ *p* < 0.05 compared with scrambled control siRNA.

We next examined whether ZEB1/2 were required for TWEAK-enhanced TGF-β1-induced EMT. BEAS-2B cells were transfected with control siRNA, ZEB1 siRNA, or ZEB2 siRNA. After incubation for 24 h, the transfected cells were treated with or without TGF-β1, TNF-α, TWEAK, or a combination of TGF-β1 and TNF-α or TWEAK for 48 h. We showed that knockdown efficiency was evaluated with quantitative real-time RT-PCR for 72 h hours (Additional file 8: Figure S8). In cells treated with ZEB2 siRNA, but not ZEB1 siRNA, up-regulation of N-cadherin mRNA expression was reduced (Figure 8). In contrast, ZEB1 and ZEB2 knockdown had no effect on E-cadherin expression (data not shown). These RNA interference assays suggested that the transcriptional repressor ZEB2 may be required for N-cadherin up-regulation in bronchial epithelial cells.

### p38 MAPK and NF-κB inhibitors inhibit TWEAK-induced mRNA expression of ZEB2 in BEAS-2B cells

**Figure 9:**
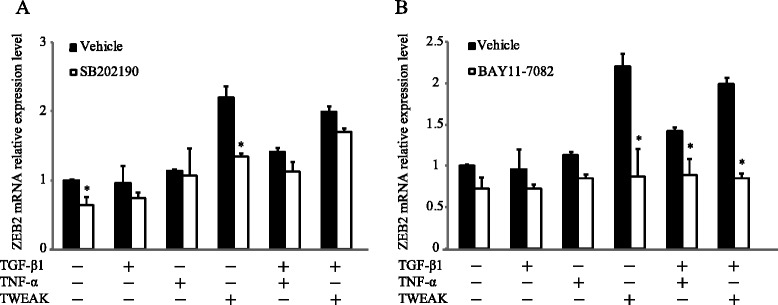
**TWEAK-induced ZEB2 mRNA expression was inhibited by inhibitors of p38 MAPK and NF-κB.** Confluent monolayers of BEAS-2B cells were cultured for 48 h in the absence (DMSO as vehicle) or presence of SB202190 (5 μM) **(A)** or BAY11-7082 (2.5 μM) **(B)** and treated with TGF-β1 (10 ng/ml), TNF-α (10 ng/ml), TWEAK (100 ng/ml), or TGF-β1 in combination with TNF-α or TWEAK, as indicated. The levels of ZEB2 mRNA were analyzed by qRT-PCR. Expression levels were normalized to the housekeeping gene GAPDH and calculated as fold induction in comparison to vehicle. Data represent the means ± SD of three independent experiments. ^*^ *p* < 0.05 compared with vehicle alone.

We finally investigated whether Smad-independent pathways, including p38 MAPK and NF-κB, were required for TWEAK-mediated mRNA expression of ZEB2. Confluent monolayers of BEAS-2B cells were cultured for 48 h with or without TGF-β1, TNF-α, TWEAK, or TGF-β1 in combination with TNF-α or TWEAK, and were treated with or without SB202190 or BAY11-7082. As shown in Figure 9, ZEB2 mRNA up-regulation induced by TWEAK was completely inhibited by BAY11-7082 and partially attenuated by SB202190. However, ZEB2 mRNA up-regulation induced by the combination of TGF-β1 and TWEAK was completely inhibited by BAY11-7082 but not by SB202190. These data suggest that at least the p38 MAPK and NF-κB pathways are required for TWEAK-induced mRNA expression of the transcriptional repressor ZEB2 in bronchial epithelial cells.

## Discussion

To our knowledge, this is the first study to show that TWEAK reduced the expression of E-cadherin, and that co-treatment with TGF-β1 and TWEAK enhanced the TGF-β1-induced features of EMT in bronchial epithelial BEAS-2B and NHBE cells. The gain of N-cadherin mesenchymal markers by the combination of TGF-β1 and TWEAK was completely abrogated by an inhibitor of the TβRI kinase signaling pathway, suggesting that TWEAK-mediated N-cadherin up-regulation requires the presence of TGF-β1. Furthermore, we also found that TWEAK accelerates TGF-β1-induced N-cadherin up-regulation via Smad-dependent and Smad-independent pathways, including p38 MAPK and NF-κB, in BEAS-2B cells. Additionally, previous reports have indicated that EMT is also regulated by a group of transcriptional repressors, including Snail, Slug, Twist, ZEB1, ZEB2, and the NF-κB transcription factor family [26]. Our experiments with ZEB2 knockdown indicated that ZEB2 might be a regulator of N-cadherin up-regulation. Moreover, we also revealed that TWEAK-induced ZEB2 mRNA expression requires p38 MAPK and NF-κB pathways. One limitation of our study was that we used monolayers of BEAS-2B and NHBE cells which show characteristics of non-differentiated basal human bronchial epithelial cells.

We recently demonstrated that EGFR transactivation is required for TGF-β enhanced epithelial wound repair [9]. In this report, although TGF-β1 and TWEAK induce the phosphorylation of EGFR, an EGFR inhibitor had no effect on EMT, suggesting that EGFR transactivation is not required for the TGF-β1-induced and TWEAK-mediated features of EMT. We also revealed that TWEAK alone and in combination with TGF-β1 induced E-cadherin down-regulation, that TWEAK had no effect on Smad2 phosphorylation, and that various inhibitors of Smad-dependent and Smad-independent signaling pathways could not suppress TWEAK-mediated E-cadherin down-regulation. Further studies are needed to investigate whether another signaling pathway or the combinations of these pathways are required for TWEAK-mediated E-cadherin down-regulation in bronchial epithelial cells, because we were not able to elucidate that mechanism.

Asthma may be accompanied by a decline in lung function despite anti-inflammatory treatment. This clinical feature has been associated with structural changes in the bronchial tissue termed airway remodeling [27,28]. The pathogenesis of airway remodeling has been previously attributed to a cycle of persistent airway epithelial damage, improper repair, and EMT, which is a complex reprogramming process of epithelial cells. Many lines of evidence imply that EMT is controlled by TGF-β1 as a major pathogenic factor. We recently reported that mechanical injury of bronchial epithelial cells induces the production of both TGF-β1 and TGF-β2, which enhances epithelial wound repair [9]. Puddicombe et al. have shown that the disruption of epithelial repair leads to enhanced release of TGF-β2 by bronchial epithelial cells [29]. Moreover, we also reported that bronchial epithelial cells produce increased amounts of IL-13 during improper wound repair [18]. Taken together, these reports indicate that improper bronchial epithelial repair causes a persistent repair phenotype with overproduction of inflammatory mediators and cytokines, including TGF-β and IL-13 which are central mediators of asthma. It is also recognized from recent studies that a number of key pro-inflammatory cytokines, including IL-1β and TNF-α, are able to enhance TGF-β-induced EMT in epithelial cells [12,13,30]. Here, we provide the first evidence, to our knowledge, that the TNF superfamily protein TWEAK accelerates the TGF-β-induced features of EMT in bronchial epithelial cells in an *in vitro* culture model. Orchestrated cellular processes with TGF-β and TWEAK in airway epithelial cells causes enhanced EMT, which may contribute to chronic airway changes and remodeling.

Our data of ECM production have suggested producing different ECM profiles of bronchial epithelial cells according to the stimulation of TGF-β, TNF-α, or TWEAK. Although the expressions of Tenascin-C and fibronectin were up-regulated by a combination with TGF-β1 and TNF-α, but not TWEAK, HAS2 expression was up-regulated by a combination with TGF-β1 and TWEAK, but not TNF-α. It was reported that TNF-α promoted CD44 expression and leading to the interaction of CD44 and hyaluronan which was synthesized by HAS [30]. Moreover, TGF-β1-induced EMT depends on HAS2 in normal mouse mammary epithelial cells [31]. Together, these results and previous reports suggest that EMT requires not only TGF-β1 and TNF-α, but also TWEAK which induced HAS2 expression.

TWEAK is a multifunctional cytokine that may promote cell death, cell proliferation, inflammation, and angiogenesis [32,33]. Also, TWEAK and its receptor, Fn14, are up-regulated in cancer, and TWEAK-induced signaling promotes multiple processes known to contribute to tumor growth [15,34,35]. However, it is not clear whether tumor metastasis requires TWEAK. Our findings show that the TWEAK-Fn14 signaling system may be a potential regulator of the TGF-β1-induced EMT that is an important component of tumor metastasis. Furthermore, TWEAK has already been evaluated as a target molecule in antitumor therapy using RG7212, an antagonistic anti-TWEAK antibody [35]. Preliminary results from a phase I study of RG7212 have reported decreased tumor cell proliferation and pathway activity plus immune cell changes in patients with advanced malignant melanoma, non-small cell lung cancer, mesothelioma, kidney cancer, and biliary tract cancer without any serious adverse side-effects [36]. Further studies are needed to assess the potential of RG7212 therapy for airway remodeling of chronic airway inflammatory disorders such as asthma and interstitial pneumonia.

## Conclusions

We have revealed a novel mechanism of EMT in bronchial epithelial cells. TWEAK induced down-regulation of E-cadherin and enhanced EMT-like phenotypic changes by TGF-β1 in bronchial epithelial cells. Furthermore, TWEAK-mediated N-cadherin up-regulation might require Smad-dependent and Smad-independent pathways, including p38 MAPK and NF-κB, and the transcriptional repressor ZEB2. These findings suggest that the pro-inflammatory cytokine TWEAK can synergize with TGF-β1 in EMT and may contribute to chronic airway changes and remodeling.

## Additional files

Additional file 1: Figure S1. Cell surface expression of Fn14 on BEAS-2B cells. After 48 hours of serum starvation, confluent monolayers of BEAS-2B cells were collected and analyzed by flow cytometry. Black and gray lines indicate marker expression and the isotype control staining, respectively. Additional file 2: Figure S2. TGF-β1 in combination with TWEAK induced E-cadherin down-regulation and N-cadherin up-regulation in a dose-dependent manner. Confluent monolayers of BEAS-2B cells were cultured for 48 h in the absence or presence of TGF-β1 (2 or 10 ng/ml) and TWEAK (100 ng/ml), as indicated. The levels of E-cadherin (A) and N-cadherin (B) mRNA were analyzed by qRT-PCR. Expression levels were normalized to the housekeeping gene GAPDH and calculated as fold induction in comparison to the control. Data represent the means ± SD of three independent experiments. ^*^ *p* < 0.05 compared with the untreated culture. ^†^ *p* < 0.05 compared with 2 ng/ml of TGF-β1. Additional file 3: Figure S3. A combination of TGF-β1 and TNF-α, but not TWEAK, enhanced TGF-β1-induced mRNA expression of Tenascin-C and fibronectin as an extracellular matrix protein. Confluent monolayers of BEAS-2B were cultured for 48 h in the absence or presence of TGF-β1 (10 ng/ml), TNF-α (10 ng/ml), TWEAK (100 ng/ml), or TGF-β1 in combination with TNF-α or TWEAK, as indicated. The levels of Tenascin-C (A) and fibronectin (B) mRNA were analyzed by qRT-PCR. Expression levels were normalized to the housekeeping gene GAPDH and calculated as fold induction in comparison to the control. Data represent the means ± SD of three independent experiments. ^*^ *p* < 0.05 compared with the untreated culture. ^†^ *p* < 0.05 compared with TGF-β1 alone. Additional file 4: Figure S4. Cell migration analysis by wound-healing assay. Confluent monolayers of BEAS-2B cells were subjected to linear injuries using a 20-μl pipette tip and then cultured in the absence (control) or presence of TGF-β1 (10 ng/ml), TNF-α (10 ng/ml), TWEAK (100 ng/ml), or TGF-β1 in combination with TNF-α or TWEAK as indicated. Photographs were taken 0 and 48 hours after wound creation. Scale bar 300 μm. Additional file 5: Figure S5. The treatment with TGF-β1, TNF-α, TWEAK, or TGF-β1 in combination with TNF-α or TWEAK had no effect on proliferation assay in BEAS-2B cells. Cells were incubated with or without TGF-β1 (10 ng/ml), TNF-α (10 ng/ml), or different concentrations of TWEAK (1-100 ng/ml) for 48 h. Cell proliferation was monitored using Cell counting Kit 8. Data represent the means ± SD of three independent experiments. Additional file 6: Figure S6. The combination with TGF-β1 and TWEAK activate Smad-independent signaling pathways. Confluent monolayers of BEAS-2B cells were cultured in the absence (DMSO as vehicle) or presence of AZD6244 (5 μM), SB202190 (5 μM), SP600125 (5 μM), LY294002 (5 μM), or BAY11-7082 (2.5 μM), AG1478 (1 μM) and treated with TGF-β1 (10 ng/ml), TNF-α (10 ng/ml), TWEAK (100 ng/ml), or TGF-β1 in combination with TNF-α or TWEAK, at different times of incubation: ERK, p38 MAPK, and p65 NF-κB at 30 min; JNK, PI3K, and EGFR at 1 h. Whole cell lysates were immunoblotted for phosphorylation of ERK (A, upper), p38 MAPK (B, upper), JNK (C, upper), PI3K (D, upper), p65 NF-κB (E, upper), and EGFR (F, upper). Densitometry of these signals was normalized against total ERK, p38 MAPK, JNK, PI3K, NF-κB, and EGFR signals, respectively (lower). Data represent the means ± SD of two independent experiments. ^*^ *p* < 0.05 compared with vehicle control. ^†^ *p* < 0.05 compared with vehicle. Additional file 7: Figure S7. SP600125, JNK inhibitor, had no apparent effect on the N-cadherin up-regulation by a combination of TGF-β1 and TWEAK. Confluent monolayers of BEAS-2B cells were cultured for 48 h in the absence (DMSO as vehicle) or presence of SP600125 (5 μM) and treated with TGF-β1, TWEAK, or TGF-β1 in combination with TNF-α or TWEAK, as indicated. Whole cell lysates were immunoblotted for N-cadherin (upper). The membranes for N-cadherin were re-probed with anti-α-tublin antibody to confirm equal loading. The density of N-cadherin was normalized with α-tubulin and quantified by densitometry (lower). Data represent the means ± SD of two independent experiments. ^*^ *p* < 0.05 compared with vehicle. Additional file 8: Figure S8 Knockdown efficiency of ZEB1 and ZEB2 mRNA expression. Total RNA was extracted from BEAS-2B cells treated with or without TGF-β1 (10 ng/ml), TNF-α (10 ng/ml), TWEAK (100 ng/ml), or TGF-β1 in combination with TNF-α or TWEAK for 48 h at 72 h after transfection with control siRNA, ZEB1 siRNA (A), or ZEB2 siRNA (B). The levels of ZEB1 and ZEB2 mRNA were analyzed by qRT-PCR. Expression levels were normalized to the housekeeping gene GAPDH and calculated as fold induction in comparison to the control. Data represent the means ± SD of three independent experiments. ^*^ *p* < 0.05 compared with scrambled control siRNA.

## Abbreviations

COPD: Chronic obstructive pulmonary disease
ECM: Extracellular matrix
EMT: Epithelial-mesenchymal transition
TGF-β: Ttransforming growth factor-β
TNF-α: Tumor necrosis factor-α
IL-1β: Interleukin-1β
TWEAK: TNF-like weak inducer of apoptosis
Fn14: Fibroblast growth factor-inducible 14
DAPI: 4′: 6-diamidino-2-phenylindole
ERK: Extracellular signal-regulated kinase
MAPK: Mitogen-activated protein kinase
JNK: Jun N-terminal kinase
PI3K: Phosphatidylinositol 3-kinase
NF-κB: Nuclear factor κB
EGFR: Epidermal growth factor receptor
