# Supplementary figures and images for: TWEAK enhances TGF-β-induced epithelial-mesenchymal transition in human bronchial epithelial cells
Source: Respir Res. 2015 Apr 8;16(1):48. doi: 10.1186/s12931-015-0207-5 (PMC4397832; doi:10.1186/s12931-015-0207-5)

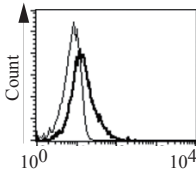

Supplement: Additional file 1: Figure S1. — Cell surface expression of Fn14 on BEAS-2B cells. After 48 hours of serum starvation, confluent monolayers of BEAS-2B cells were collected and analyzed by flow cytometry. Black and gray lines indicate marker expression and the isotype control staining, respectively. [file 12931_2015_207_MOESM1_ESM.pdf]

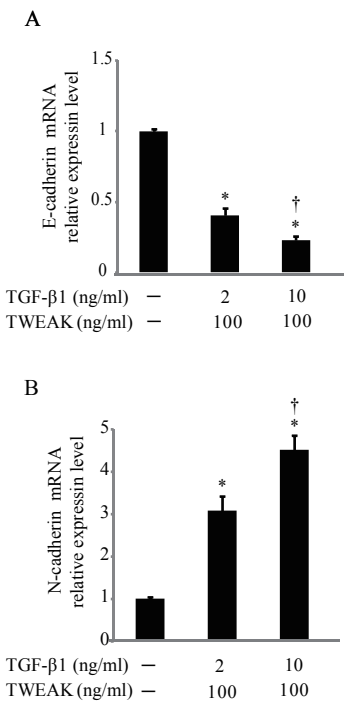

Supplement: Additional file 2: Figure S2. — TGF-β1 in combination with TWEAK induced E-cadherin down-regulation and N-cadherin up-regulation in a dose-dependent manner. Confluent monolayers of BEAS-2B cells were cultured for 48 h in the absence or presence of TGF-β1 (2 or 10 ng/ml) and TWEAK (100 ng/ml), as indicated. The levels of E-cadherin (A) and N-cadherin (B) mRNA were analyzed by qRT-PCR. Expression levels were normalized to the housekeeping gene GAPDH and calculated as fold induction in comparison to the control. Data represent the means ± SD of three independent experiments. * p < 0.05 compared with the untreated culture. † p < 0.05 compared with 2 ng/ml of TGF-β1. [file 12931_2015_207_MOESM2_ESM.pdf]

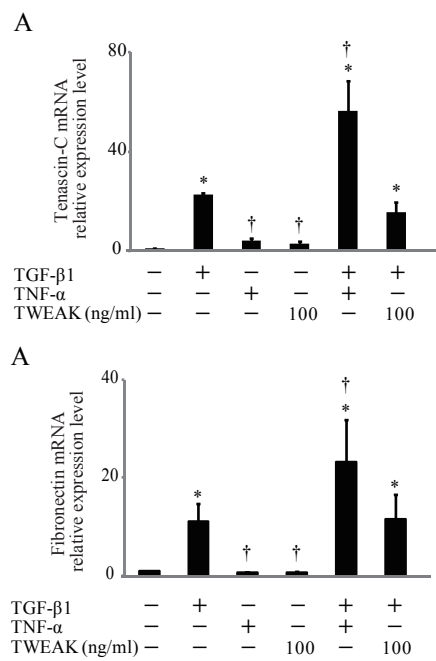

Supplement: Additional file 3: Figure S3. — A combination of TGF-β1 and TNF-α, but not TWEAK, enhanced TGF-β1-induced mRNA expression of Tenascin-C and fibronectin as an extracellular matrix protein. Confluent monolayers of BEAS-2B were cultured for 48 h in the absence or presence of TGF-β1 (10 ng/ml), TNF-α (10 ng/ml), TWEAK (100 ng/ml), or TGF-β1 in combination with TNF-α or TWEAK, as indicated. The levels of Tenascin-C (A) and fibronectin (B) mRNA were analyzed by qRT-PCR. Expression levels were normalized to the housekeeping gene GAPDH and calculated as fold induction in comparison to the control. Data represent the means ± SD of three independent experiments. * p < 0.05 compared with the untreated culture. † p < 0.05 compared with TGF-β1 alone. [file 12931_2015_207_MOESM3_ESM.pdf]

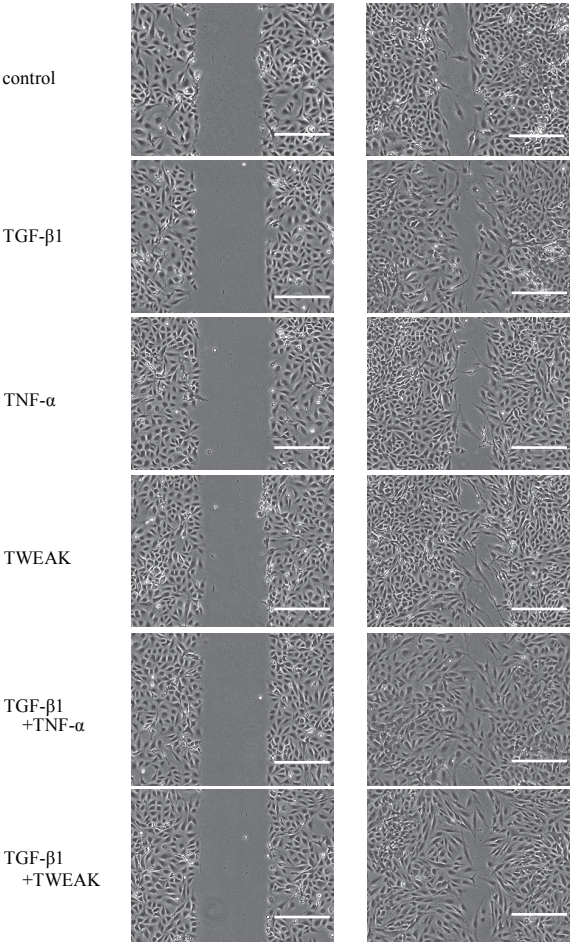

Supplement: Additional file 4: Figure S4. — Cell migration analysis by wound-healing assay. Confluent monolayers of BEAS-2B cells were subjected to linear injuries using a 20-μl pipette tip and then cultured in the absence (control) or presence of TGF-β1 (10 ng/ml), TNF-α (10 ng/ml), TWEAK (100 ng/ml), or TGF-β1 in combination with TNF-α or TWEAK as indicated. Photographs were taken 0 and 48 hours after wound creation. Scale bar 300 μm. [file 12931_2015_207_MOESM4_ESM.pdf]

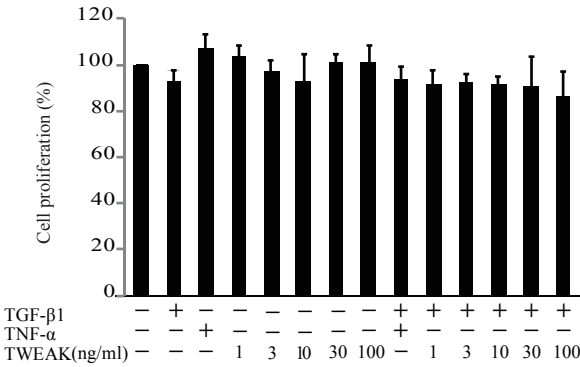

Supplement: Additional file 5: Figure S5. — The treatment with TGF-β1, TNF-α, TWEAK, or TGF-β1 in combination with TNF-α or TWEAK had no effect on proliferation assay in BEAS-2B cells. Cells were incubated with or without TGF-β1 (10 ng/ml), TNF-α (10 ng/ml), or different concentrations of TWEAK (1-100 ng/ml) for 48 h. Cell proliferation was monitored using Cell counting Kit 8. Data represent the means ± SD of three independent experiments. [file 12931_2015_207_MOESM5_ESM.pdf]

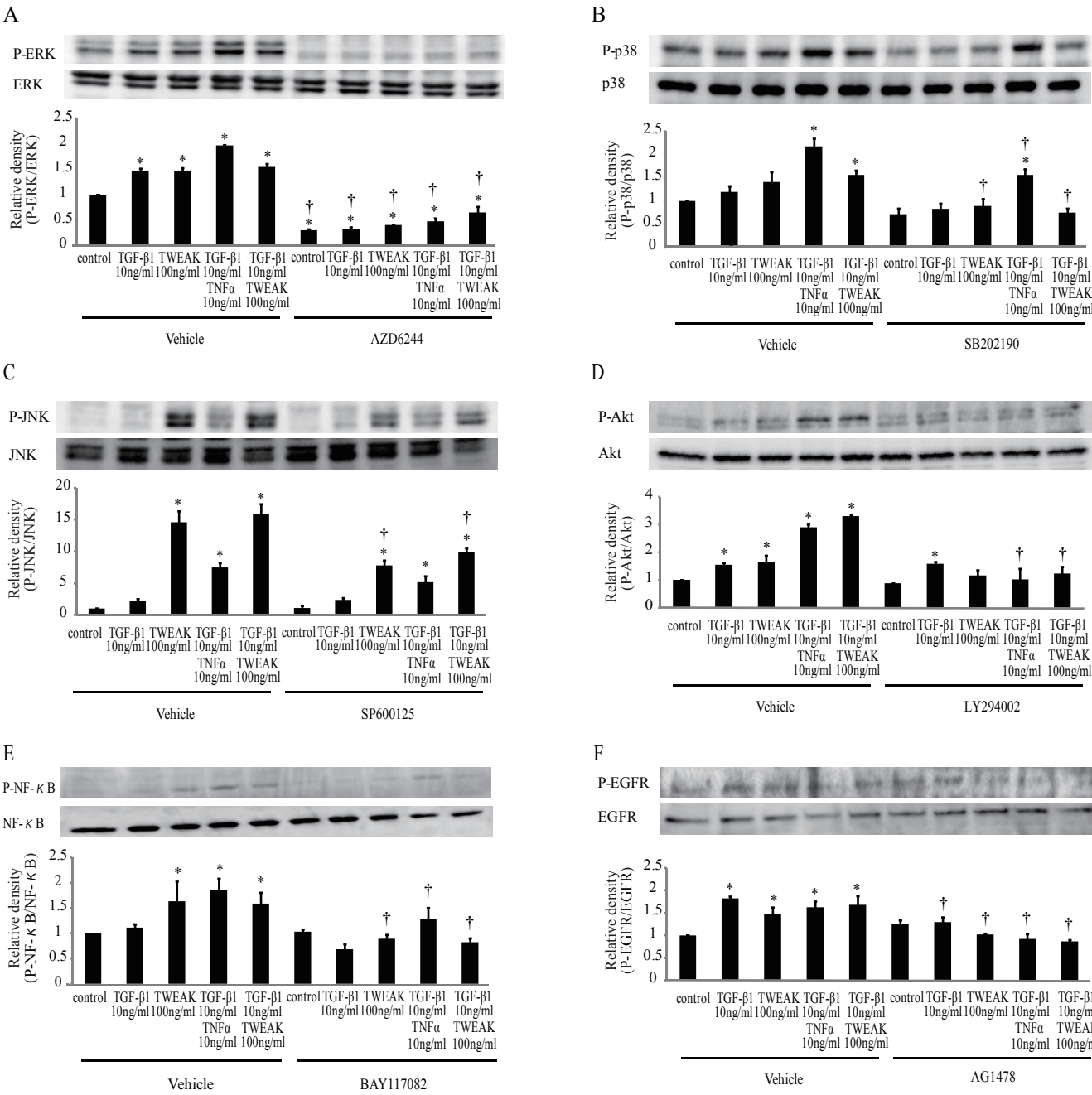

Supplement: Additional file 6: Figure S6. — The combination with TGF-β1 and TWEAK activate Smad-independent signaling pathways. Confluent monolayers of BEAS-2B cells were cultured in the absence (DMSO as vehicle) or presence of AZD6244 (5 μM), SB202190 (5 μM), SP600125 (5 μM), LY294002 (5 μM), or BAY11-7082 (2.5 μM), AG1478 (1 μM) and treated with TGF-β1 (10 ng/ml), TNF-α (10 ng/ml), TWEAK (100 ng/ml), or TGF-β1 in combination with TNF-α or TWEAK, at different times of incubation: ERK, p38 MAPK, and p65 NF-κB at 30 min; JNK, PI3K, and EGFR at 1 h. Whole cell lysates were immunoblotted for phosphorylation of ERK (A, upper), p38 MAPK (B, upper), JNK (C, upper), PI3K (D, upper), p65 NF-κB (E, upper), and EGFR (F, upper). Densitometry of these signals was normalized against total ERK, p38 MAPK, JNK, PI3K, NF-κB, and EGFR signals, respectively (lower). Data represent the means ± SD of two independent experiments. * p < 0.05 compared with vehicle control. † p < 0.05 compared with vehicle. [file 12931_2015_207_MOESM6_ESM.pdf]

Supplemental Figure S7

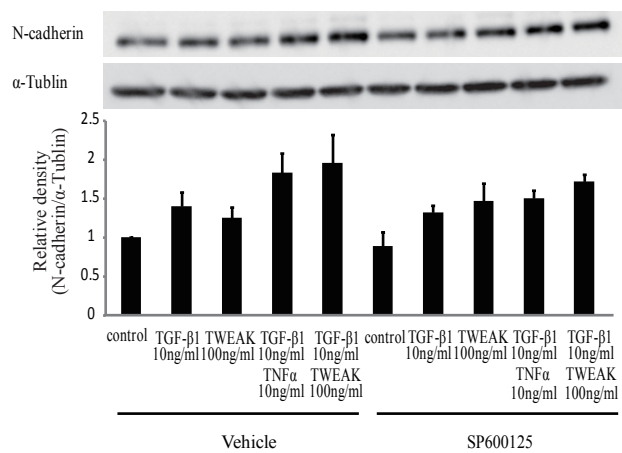

Supplement: Additional file 7: Figure S7. — SP600125, JNK inhibitor, had no apparent effect on the N-cadherin up-regulation by a combination of TGF-β1 and TWEAK. Confluent monolayers of BEAS-2B cells were cultured for 48 h in the absence (DMSO as vehicle) or presence of SP600125 (5 μM) and treated with TGF-β1, TWEAK, or TGF-β1 in combination with TNF-α or TWEAK, as indicated. Whole cell lysates were immunoblotted for N-cadherin (upper). The membranes for N-cadherin were re-probed with anti-α-tublin antibody to confirm equal loading. The density of N-cadherin was normalized with α-tubulin and quantified by densitometry (lower). Data represent the means ± SD of two independent experiments. * p < 0.05 compared with vehicle. [file 12931_2015_207_MOESM7_ESM.pdf]

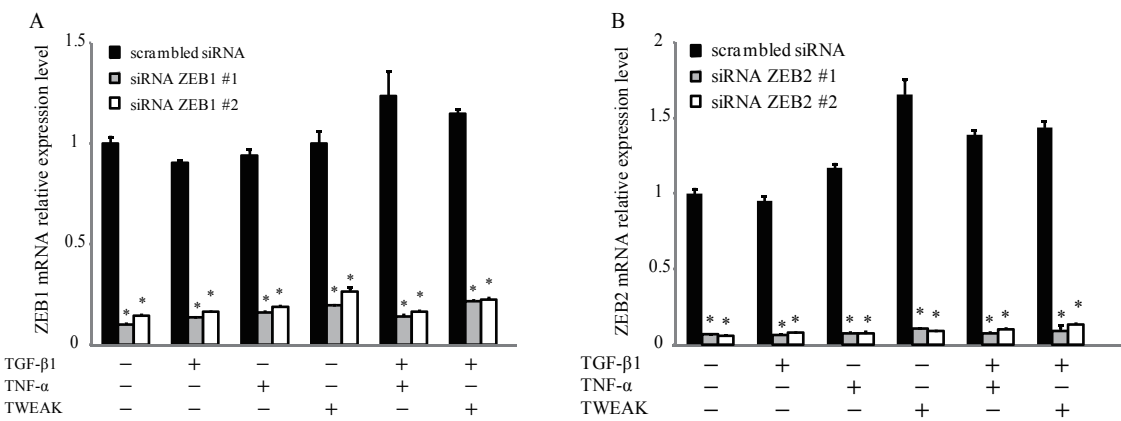

Supplement: Additional file 8: Figure S8 — Knockdown efficiency of ZEB1 and ZEB2 mRNA expression. Total RNA was extracted from BEAS-2B cells treated with or without TGF-β1 (10 ng/ml), TNF-α (10 ng/ml), TWEAK (100 ng/ml), or TGF-β1 in combination with TNF-α or TWEAK for 48 h at 72 h after transfection with control siRNA, ZEB1 siRNA (A), or ZEB2 siRNA (B). The levels of ZEB1 and ZEB2 mRNA were analyzed by qRT-PCR. Expression levels were normalized to the housekeeping gene GAPDH and calculated as fold induction in comparison to the control. Data represent the means ± SD of three independent experiments. * p < 0.05 compared with scrambled control siRNA. [file 12931_2015_207_MOESM8_ESM.pdf]
